# 

**Published:** 1902-12

**Authors:** 


### BOOKS RECEIVED.

Practical Gynecology, Obstetrics, and the Menopause. By A. H. P. Leuf, M. D., Philadelphia. Three Parts, complete in one volume of 326 pages. Published by the Medical Council, Philadelphia, Pa. 1902. [Price, $2.50.]

The Practical Medicine Series of Year Books. Ten volumes. Issued monthly under the general editorial charge of Gustavus P. Head, M. D., Professor of Laryngology and Rhinology in the Chicago Post-Graduate Medical School. Volume I. General Medicine. Edited by Frank Billings, M. S., M. D, Head of the Medical Department and Dean of the Faculty of Rush Medical College, Chicago, and J. H. Salisbury, M. D., Professor of Medicine, Chicago Clinical School. 12 mo, pp. 358. Chicago: The Year Book Publishers. 1902. [Price, $1.50. Entire series, $7.50.]

Spectacles aud Eyeglasses. Their Forms, Mounting and Proper Adjustment. By R. J. Phillips, M. D., Ophthalmologist Presbyterian Orphanage, Philadelphia. Third edition revised. With 52 illustrations. Philadelphia: P. Blakiston's Son & Co. 1902. [Price, $1.00.]

International Clinics. A Quarterly of Illustrated Clinical Lectures and especially prepared Articles on Medicine, Neurology, Surgery, Therapeutics, Obstetrics, Pediatrics, Pathology, Dermatology, Diseases of the Eye, Ear, Nose, and Throat, and other Topics of Interest to Students and Practitioners by leading Members of the Medical Profession throughout the World. Edited by Henry W. Cattell, A. M., M D., Philadelphia, with the Collaboration of John B. Murphy, M. D., Chicago; Alexander D. Blackader, M. D., Montreal; H. C. Wood, M. D., Philadelphia; T. M. Rotch, M. D., Boston; E. Landolt, M. D., Paris; Thomas G. Morton, M. D., Philadelphia; James J. Walsh, M. D., New York; J. W. Ballantyne, M. D., Edinburgh, and John Harold, M. D, London, with Regular Correspondents in Montreal, London, Paris, Leipsic, and Vienna. J. B. Lippincott Company, Philadelphia and London. [Cloth, $2.00.] Volume III. Twelfth Series, 1902.]

A Treatise on Massage. Its History, Mode of Application and Effects, Indications and Contraindications. By Douglas Graham, M. D., Boston. Third edition revised, enlarged and illustrated. Octavo, pp. 462. Philadelphia and London: J. B. Lippincott Company. 1902. [Price, $4.00.]

A Handbook of Materia Medica, Pharmacy and Therapeutics, including the Physiological Action of Drugs, the Special Therapeutics of Disease, Official and Practical Pharmacy, and minute directions for prescription writing. By Samuel O. L. Potter, A. M., M. D., M. R., C. P. Lond, formerly Professor of the Principles and Practice of Medicine in the Cooper Medical College of San Francisco. Ninth edition revised and enlarged. Octavo, pp. 951. Philadelphia: P. Blakiston's Son & Co. 1902. [Price, $5.00 net.]

The American Textbook of Obstetrics. In two volumes. Edited by Richard C. Norris, M. D.; Art Editor, Robert L. Dickinson, M. D. Second edition, thoroughly revised and enlarged. Two handsome imperial octavo volumes of about 600 pages each; nearly 600 text illustrations, and 49 colored and half-tone plates. Philadelphia and London: W. B. Saunders & Co., 1902. [Per volume: cloth, $3.50 net; sheep or half morocco, $4.00 net.]

American Edition of Nothnagel's Practice. Volume IV. Diseases of the Bronchi and Lungs. Diseases of the Bronchi. By Dr. F. A. Hoffman, of Leipsic. Diseases of the Pleura. By Dr. O. Roseebach, of Berlin. Pneumonia. By Dr. F. Aufrecht, of Magdeburg. Edited, with additions, by John H. Musser, M. D., Professor of Clinical Medicine, University of Pennsylvania. Handsome octavo volume of 1030 pages, illustrated, including 7 full page colored lithographic plates. Philadelphia and London: W. B. Saunders & Co. 1902. [Cloth, $5.00 net; half morocco, $6.00 net.]

The Development of the Human Body. A Manual of Human Embryology. By J. Playfair McMurrich, A. M., Ph. D., Professor of Anatomy in the University of Michigan. 12 mo, pp. 573. With 270 illustrations. Philadelphia: P. Blakiston's Sons & Co. 1902. [Price, $3.00.]

A Text Book of Diseases of the Eye. A Handbook of Ophthalmic Practice for Students and Practitioners. By G. E. De Schweinitz, A. M., M. D., Professor of Ophthalmology in the University of Pennsylvania, etc. Fourth edition, revised, enlarged, and entirely reset. Octavo volume of 773 pages, with 280 text-illustrations and 6 chromo-lithographic plates. Philadelphia and London: W. B. Saunders & Co. 1902. [Cloth, $5.00 net; sheep or half morocco, $6.00 net.]

Bacteriological Technique. A Laboratory Guide for the Medical, Dental, and Technical Student. By. J W. H. Eyre, M. D., F. R. S., Edin., Bacteriologist to Guy's Hospital, and Lecturer on Bacteriology at the Medical and Dental Schools, etc. Octavo of 375 pages, with 170 illustrations. Philadelphia and London: W. B. Saunders & Co. 1902. [Cloth, $2.50 net.]

A Treatise on Diseases of the Eye, Nose, Throat, and Ear. For Students and Practitioners. By various authors. Edited by William Campbell Posey, A. B., M. D., Professor of Ophthalmology in the Philadelphia Polyclinic; Surgeon to the Wills Eye Hospital; Ophthalmic Surgeon to the Howard and Epileptic Hospitals. And Jonathan Wright, M. D., Attending Laryngologist to Kings County Hospital, Brooklyn. Royal octavo, pp. 1252. Illustrated with 650 engravings and 35 plates in colors and monochrome. Lea Brothers & Co., Philadelphia and New York. 1902. [Cloth, $7.00; leather, $8 00.]

Clinical Surgery, for the Instruction of Practitioners and Students of Surgery. By A. J. Ochsner, B. S., F. R. M. S., M. D., Chicago, Surgeon-in-Chief, Augustana Hospital, and St. Mary's Hospital; Professor of Clinical Surgery, Medical Department, University of Illinois. Royal Octavo, pp. 481. Profusely illustrated. Cleveland Press (The Clinical Review Publishing Company). 1902. [Price: cloth, $6.00; half morocco, $7.00.]

Memoranda on Poisons, by Thomas Tanner, M. D., F. L. S. Ninth revised edition, by Henry Leffmann, A. M., M. D., Professor of Chemistry in the Woman's Medical College of Pennsylvania. Philadelphia: P. Blakiston's Son & Co. 1902. [Price, 75 cents.]

The Public and the Doctor. By B. E. Hadra, M. D., Dallas, Texas. 1902. [Price, 50 cents.]

The Medical Epitomé Series. Diseases of the Skin. A Manual for Students and Practitioners. By Alfred Schalek, M. D, instructor of Dermatology, Genito-urinary, and Venereal Diseases, Rush Medical College, Chicago. Series edited by V. C. Pedersen, A. M., M. D., House Surgeon at the New York Hospital. Illustrated with 34 engravings. Lea Brothers & Co., Philadelphia and New York. 1902. [Price, $1.00.]

The Practice of Surgery. A Treatise on Surgery for the Use of Practitioners and Students. By Henry R. Wharton, M. D., Clinical Professor of Surgery, Woman's Medical College of Pennsylvania; Surgeon to the Presbyterian and Children's Hospitals, Philadelphia, and B. Farquhar Curtis, M. D., Professor of Clinical Surgery, and Adjunct Professor of the Principles of Surgery in the University and Bellevue College of New York. Third edition. Octavo, pp. 1249. Illustrated, Philadelphia and London: J. B. Lippincott Company. 1902.

A Textbook of Pathology, and Pathological Anatomy. By Dr. Hans Schmaus, Extraordinary Professor, and First Assistant in the Pathological Institute, Munich. Translated from the sixth German edition by A. E. Thayer, M. D., Instructor in Pathology in Cornell University Medical College, New York. Edited with additions by James Ewing, M. D., Professor of Pathology in Cornell University Medical College, New York. Octavo, pp. 614. Illustrated with 351 engravings, including 35 colored insert plates. Lea Brothers & Co., Philadelphia and New York. 1902. [Cloth, $4.00 net.]

A Textbook of the Science and Art of Obstetrics. By Henry J. Garrigues, A. M., M. D. Consulting Obstetric Surgeon to the New York Maternity Hospital, Gynecologist to St. Mark's Hospital. Octavo, pp. 874. 504 Illustrations. Philadelphia and London: J. B. Lippincott Co. 1902.

